# Infections in the first year of life and development of beta cell autoimmunity and clinical type 1 diabetes in high-risk individuals: the TRIGR cohort

**DOI:** 10.1007/s00125-022-05786-3

**Published:** 2022-09-09

**Authors:** Olga Kordonouri, David Cuthbertson, Malin Belteky, Bärbel Aschemeier-Fuchs, Neil H. White, Elisabeth Cummings, Mikael Knip, Johnny Ludvigsson

**Affiliations:** 1 Children’s Hospital Auf Der Bult, Hannover Medical School, Hannover, Germany; 2 Health Informatics Institute, University of South Florida, Tampa, FL USA; 3 Crown Princess Victoria Children’s Hospital and Division of Pediatrics, Department of Biomedical and Clinical Sciences, Linköping University, Linköping, Sweden; 4 Department of Pediatrics, Washington University in St Louis, St Louis, MO USA; 5 Department of Pediatrics IWK Health/Dalhousie University, Halifax, NS Canada; 6 Pediatric Research Center, New Children’s Hospital, Helsinki University Hospital, Helsinki, Finland; 7 Research Program for Clinical and Molecular Metabolism, Faculty of Medicine, University of Helsinki, Helsinki, Finland; 8 Tampere Center for Child Health Research, Tampere University Hospital, Tampere, Finland

**Keywords:** Autoimmunity, Children, Early infections, TRIGR, Type 1 diabetes

## Abstract

**Aims/hypothesis:**

Accumulated data suggest that infections in early life contribute to the development of type 1 diabetes. Using data from the Trial to Reduce IDDM in the Genetically at Risk (TRIGR), we set out to assess whether children who later developed diabetes-related autoantibodies and/or clinical type 1 diabetes had different exposure to infections early in life compared with those who did not.

**Methods:**

A cohort of 2159 children with an affected first-degree relative and HLA-conferred susceptibility to type 1 diabetes were recruited between 2002 and 2007 and followed until 2017. Infections were registered prospectively. The relationship between infections in the first year of life and the development of autoantibodies or clinical type 1 diabetes was analysed using univariable and multivariable Cox regression models. As this study was exploratory, no adjustment was made for multiple comparisons.

**Results:**

Adjusting for HLA, sex, breastfeeding duration and birth order, those who had seven or more infections during their first year of life were more likely to develop at least one positive type 1 diabetes-related autoantibody (*p*=0.028, HR 9.166 [95% CI 1.277, 65.81]) compared with those who had no infections. Those who had their first viral infection aged between 6 and 12 months were less likely to develop at least one positive type 1 diabetes-related antibody (*p*=0.043, HR 0.828 [95% CI 0.690, 0.994]) or multiple antibodies (*p*=0.0351, HR 0.664 [95% CI 0.453, 0.972]). Those who had ever had an unspecified bacterial infection were more likely to develop at least one positive type 1 diabetes-related autoantibody (*p*=0.013, HR 1.412 [95% CI 1.075, 1.854]), to develop multiple antibodies (*p*=0.037, HR 1.652 [95% CI 1.030, 2.649]) and to develop clinical type 1 diabetes (*p*=0.011, HR 2.066 [95% CI 1.182, 3.613]).

**Conclusions/interpretation:**

We found weak support for the assumption that viral infections early in life may initiate the autoimmune process or later development of type 1 diabetes. In contrast, certain bacterial infections appeared to increase the risk of both multiple autoantibodies and clinical type 1 diabetes.

**Graphical abstract:**

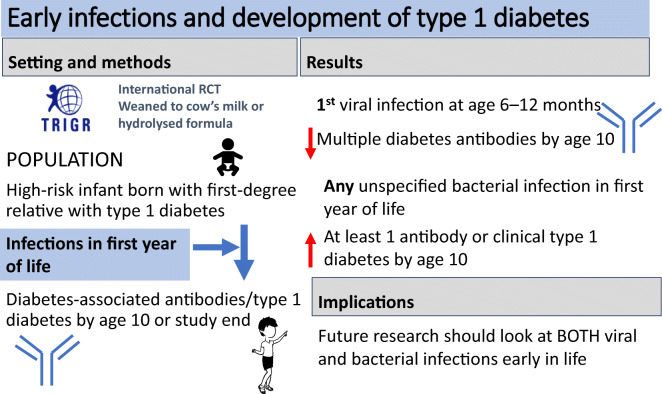

**Supplementary Information:**

The online version of this article (10.1007/s00125-022-05786-3) contains peer-reviewed but unedited supplementary material.

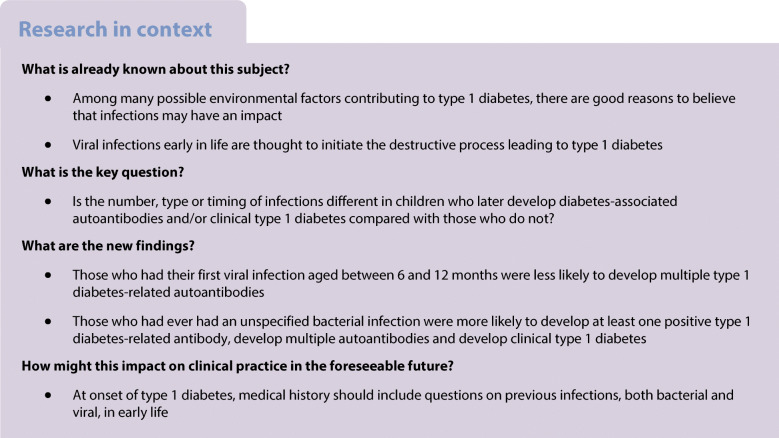



## Introduction

Despite modern treatment, type 1 diabetes is a serious disease, with increased morbidity and mortality risk even in patients with good metabolic control [1]. Even in countries with the best available insulin and modern devices, life expectancy may be shortened by several years [2]. The disease cannot be cured, and the incidence has been increasing in many countries around the world [3]. Even though it may theoretically be possible to delay the onset of the disease a few years by treating very high-risk individuals with anti-CD3 monoclonal antibodies before the disease manifests [4], such intervention could only be an option in a very limited group of individuals, and is associated with both adverse events and risks.

For primary prevention, it is necessary to identify the so far unknown cause or causes of the disease. Genetic predisposition is significant [5], but several facts strongly indicate that environmental and/or lifestyle factors play an important role in addition to genetic factors [6]. The incidence of type 1 diabetes has increased significantly over the last few decades [3], which cannot be explained by genetic factors. The impact of environmental factors is supported by the remarkable difference in type 1 diabetes incidence between the Finnish and Russian parts of the Karelia region, whose populations share similar genetics [7], and the finding that migrants tend to show an incidence of type 1 diabetes that is similar to that of the population of their new country [8, 9]. The obvious role of lifestyle or environmental factors is in a way encouraging, as such factors may be modifiable, making it possible not only to stop the increasing incidence but actually to bend the incidence curve downwards again.

Among the many possible environmental factors contributing to type 1 diabetes [6], there is evidence suggesting that infections may have an impact. Epidemiological studies first indicated such a role [10]. Then, when a convincing case history was published 40 years ago showing that Coxsackie virus infection can cause the disease [11], the mystery appeared to be solved. However, unfortunately this is still not the case. We know that maternal infections during pregnancy appear to be associated with type 1 diabetes in the offspring [12], and it has been reported that children who later develop type 1 diabetes more often had infections early in life [13, 14]. Many studies have pointed to enterovirus infections [15–17]. It has been suggested that the infections may initiate the autoimmune process [18–20], but another report suggested that such an infection may accelerate the disease process [21]. Studies also support the importance of infections in precipitating manifest disease, such as increased levels of IgM virus antibodies at the time of diagnosis of type 1 diabetes [22], and the clustering of diagnoses of type 1 diabetes in both time and space [23]. Infectious agents may attack the pancreas, and signs of persistent virus have been detected in the pancreas of patients with newly diagnosed type 1 diabetes [24]. Infections may also influence the immune balance and cause increased insulin demand, which may contribute to manifestation of the disease [25]. It is mostly enteroviruses that have been implicated, but recent studies suggest that upper respiratory tract infections may also be involved [26, 27]. However, despite these supporting pieces of evidence, the picture is far from clear [28]. Many different viruses may play a role as initiating, accelerating and precipitating factors [12]. Bacteria may also be involved, not least via the gut microbiota [29]. Therefore, it is likely that infections during early childhood play a role in the development of type 1 diabetes, but it has been challenging to identify any clear patterns [30]. In an effort to further elucidate the role of such infections, we have used prospectively collected data from the Trial to Reduce IDDM in the Genetically at Risk (TRIGR) study [31]. In this trial, infections were prospectively recorded in a large number of children with genetic susceptibility to type 1 diabetes who were followed from birth until at least the age of 10 years or until diagnosis of type 1 diabetes. We assessed whether the number, type or timing of infections in the first year of life are different in children who later develop diabetes-associated autoantibodies and/or clinical type 1 diabetes compared with those who do not.

## Methods

The TRIGR study was an international double-blind multicentre trial designed to determine whether weaning to a hydrolysed infant formula compared with a cow’s milk-based formula reduced the incidence of diabetes-associated autoantibodies and clinical type 1 diabetes in children with an affected first-degree relative and HLA-conferred increased disease susceptibility [29]. A total of 2159 participants were recruited between May 2002 and January 2007, and followed until February 2017 when all children were at least 10 years old [32]. Infections were documented at each participating centre at 12, 18 and 24 months old and annually thereafter during the total duration of follow-up or until the diagnosis of type 1 diabetes. As our aim is to investigate the possible importance of early infections in the first year of life, and whether infections are associated with development of autoantibodies and/or type 1 diabetes, we restricted our study to the group of children who had not developed autoantibodies and/or type 1 diabetes before 12 months old, and who had documented infection data.

None of the participants developed type 2 diabetes, and none who developed type 1 diabetes were autoantibody-negative. Islet cell antibodies (ICA) were detected by use of indirect immunofluorescence in the Scientific Laboratory, Department of Pediatrics, University of Oulu, Oulu, Finland. The disease sensitivity and specificity of the islet cell antibody assay were 100% and 98%, respectively, in the fourth round of the international workshops on standardisation of the islet cell antibody assay [33]. Other diabetes-associated autoantibodies were analysed in a central laboratory (Scientific Laboratory, Children’s Hospital, University of Helsinki, Helsinki, Finland) using samples taken at birth, at 3, 6, 9, 18 and 24 months old, and then annually. Autoantibodies to GAD (GADA), tyrosine phosphatase-related insulinoma-associated 2 molecule (IA-2A), insulin (IAA) and zinc transporter 8 (ZnT8A) were analysed using specific radiobinding immunoassays [34]. The reported disease-specific sensitivity and specificity for each autoantibody were as follows: GADA sensitivity 70–92%, specificity 90–98%; IA-2A sensitivity 62–80%, specificity 93–100%; IAA sensitivity 42–62%, specificity 93–99% [35]. The disease-specific sensitivity and specificity for ZnT8A were 62–74% and 100%, respectively. Type 1 diabetes was diagnosed according to the WHO criteria [36].

Infections as reported by parents were recorded on a standardised adverse event form. The infections were classified as follows: (1) upper respiratory infection, (2) gastroenteritis, (3) urinary tract infection, (4) middle ear infection, (5) pneumonia and (6) other infection.

When infections were categorised as ‘other’, the trial staff were required to specify details in a free text field. Data were also collected on any drug treatments, with antibiotics as a specific category, including the duration of therapy. Based on the clinical description and antibiotic use, infections were classified as bacterial or viral. Infections categorised as ‘other bacterial infections’ are subsequently referred to as ‘unspecified bacterial infections’.

The TRIGR study was approved by the research ethics boards/committees in all participating countries. Parents had given their informed consent, and children provided assent when appropriate for age according to local guidelines. An online repository for TRIGR data is currently not available. The code is maintained at the TRIGR Data Management Unit, University of South Florida. Information is available upon reasonable request from the Data Management Unit.

### Statistics

Participant characteristics and variables associated with infections reported during the first year were compared based on antibody status (development of any antibody or multiple [two or more] positive antibodies) and development of type 1 diabetes during follow-up. Categorical variables were compared using the χ^2^ test unless the number of observations in an individual cell was less than 10, then the Fisher’s exact test was utilised. Continuous variables were compared using the Wilcoxon rank sum test. Univariable Cox regression models based on time to development of any antibody, multiple antibodies and type 1 diabetes were developed using the participant characteristics and infection variables. Multivariable Cox regression models were then applied to adjust for HLA status, sex, breastfeeding duration and birth order. As this study was exploratory, no adjustment was made for multiple comparisons. A *p* value < 0.05 was considered significant. The data were analysed using SAS software version 9.4 (SAS Institute, USA).

## Results

Of those enrolled in TRIGR (*n*=2159), 2017 were antibody-negative and free of type 1 diabetes at 12 months and had available infection data. Table 1 summarises the characteristics of the study group by antibody and diabetes status. Of the 2017 participants included in this study, 842 (41.7%) developed at least one type 1 diabetes-related antibody, 236 (11.7%) developed multiple antibodies and 134 (6.6%) developed clinical type 1 diabetes. Among the 2017 participants, 537 (26.6%) had no recorded infections, 969 (48.0%) had 1–3 infections, 379 (18.8%) 4–6 infections and 132 (6.5%) had seven or more infections during the first year of life (ESM Table 1). The median (IQR) number of infections per participant was 2 (0–4), with the participants experiencing more viral than bacterial infections: 1 viral infection (0–3) vs 0 bacterial infections (0–1). Of the 2017 participants, 981 (48.6%) had 1–3 viral infections, 248 (12.3%) had 4–6 viral infections and 50 (2.5%) had seven or more viral infections. The incidence of infections increased with increasing age of the infants: 0.41 ± 0.78 up to 3 months, 0.54 ± 0.91 between 3 and 6 months and 1.40 ± 1.74 between 6 and 12 months old (means ± SD). Utilising univariable ANOVAs, the number of infections was related to sex (*p*=0.034) but not HLA genotype (*p*=0.302).

**Table 1:** Characteristics of participants by antibody and type 1 diabetes status

Characteristic	AB+ status			Multi-AB+ status			T1D status		
	No AB+ (*n*=1175)	Any AB+ (*n*=842)	*p* value	Not multi-AB+ (*n*=1781)	Multi-AB+ (*n*=236)	*p* value	Did not develop T1D (*n*=1883)	Developed T1D (*n*=134)	*p* value
HLA									
HLA-DQB1*0302/DQB1*02	241 (20.5)	227 (27.0)	< 0.001	387 (21.7)	81 (34.3)	< 0.001	416 (22.1)	52 (38.8)	< 0.001
HLA-DQB1*0302/x	521 (44.3)	369 (43.8)		791 (44.4)	99 (41.9)		839 (44.6)	51 (38.1)	
HLA-DQA1*05-DQB1*02/y or HLA-DQA1*03-DQB1*02/y	413 (35.1)	246 (29.2)		603 (33.9)	56 (23.7)		628 (33.4)	31 (23.1)	
Sex									
Female	569 (48.4)	393 (46.7)	0.438	854 (48.0)	108 (45.8)	0.527	893 (47.4)	69 (51.5)	0.362
Male	606 (51.6)	449 (53.3)		927 (52.0)	128 (54.2)		990 (52.6)	65 (48.5)	
Breastfeeding duration (months)									
None	27 (2.3)	20 (2.4)	0.426	40 (2.2)	7 (3.0)	0.288	43 (2.3)	4 (3.0)	0.528
0 to < 3	914 (77.8)	641 (76.1)		1384 (77.7)	171 (72.5)		1453 (77.2)	102 (76.1)	
3 to < 6	212 (18.0)	156 (18.5)		318 (17.9)	50 (21.2)		345 (18.3)	23 (17.2)	
6–9	22 (1.9)	25 (3.0)		39 (2.2)	8 (3.4)		42 (2.2)	5 (3.7)	
Birth order									
1st	627 (53.4)	445 (52.9)	0.248	959 (53.8)	113 (47.9)	0.203	1007 (53.5)	65 (48.5)	0.077
2nd	356 (30.3)	237 (28.1)		518 (29.1)	75 (31.8)		557 (29.6)	36 (26.9)	
3rd or more	192 (16.3)	160 (19.0)		304 (17.1)	48 (20.3)		319 (16.9)	33 (24.6)	

Values are *n* (%)

*p* values were calculated using χ^2^ test unless the number of observations in an individual cell was less than 10, then the Fisher’s exact test was utilised

AB+, antibody-positive; T1D, type 1 diabetes

Interestingly, among the 47 infants with no breastfeeding, 28 (59.6%) had no recorded infection during the first year of life and none had seven or more infections; among the 1970 breastfed infants, only 509 (25.8%) had no recorded infection and 132 (6.7%) had seven or more infections (*p*<0.001). Furthermore, among the breastfed infants, 36.0% had no viral infection recorded in the first year of life, but 59.6% of those who were not breastfed had no viral infection recorded (*p*<0.004).

Table 2 summarises the number and timing of infections, and Table 3 summarises the types of infections that were reported. In univariable analysis, those who developed at least one positive type 1 diabetes-related autoantibody had more infections (*p*=0.002) during the first year of life, had more bacterial infections (*p*=0.005) and had more viral infections (*p*=0.015). They were more likely to have more infections during the first 3 months of life (*p*=0.007), and more between months 6 and 12 (*p*=0.023). They were also more likely to have had an unspecified bacterial infection (*p*=0.006), more likely to have had an upper respiratory infection (*p*=0.044) and more likely to have used antibiotics (*p*=0.013). Autoantibody-positive children were more likely to have experienced their first infection earlier (*p*=0.021) and their first bacterial infection earlier (*p*=0.006). Children with multiple autoantibodies more often had any unspecified bacterial infection (*p*<0.026) and more infections at < 3 months old (*p*<0.039). Those who developed clinical type 1 diabetes were more likely to have had an unspecified bacterial infection (*p*=0.003) and to have had an unspecified viral infection (*p*=0.048) (Table 3). The Kaplan–Meier diagrams in electronic supplementary material (ESM) Figs 1–3 provide further details.

**Table 2:** Timing and number of bacterial and viral infections in the first 12 months of life by antibody and diabetes status

Infection number/timing	AB+ status			Multi-AB+ status			T1D status		
	No AB+ (*n*=1175)	Any AB+ (*n*=842)	*p* value	Not multi-AB+ (*n*=1781)	Multi-AB+ (*n*=236)	*p* value	Did not develop T1D (*n*=1883)	Developed T1D (*n*=134)	*p* value
Age at first infection (months)									
None	338 (28.8)	199 (23.6)	0.021	474 (26.6)	63 (26.7)	0.218	498 (26.4)	39 (29.1)	0.320
0 to < 3	319 (27.1)	274 (32.5)		511 (28.7)	82 (34.7)		547 (29.0)	46 (34.3)	
3 to < 6	251 (21.4)	177 (21.0)		383 (21.5)	45 (19.1)		406 (21.6)	22 (16.4)	
6–12	267 (22.7)	192 (22.8)		413 (23.2)	46 (19.5)		432 (22.9)	27 (20.1)	
Age at first viral infection									
None	448 (38.1)	290 (34.4)	0.197	656 (36.8)	82 (34.7)	0.663	690 (36.6)	48 (35.8)	0.992
0 to < 3	235 (20.0)	186 (22.1)		369 (20.7)	52 (22.0)		393 (20.9)	28 (20.9)	
3 to < 6	213 (18.1)	174 (20.7)		336 (18.9)	51 (21.6)		360 (19.1)	27 (20.1)	
6–12	279 (23.7)	192 (22.8)		420 (23.6)	51 (21.6)		440 (23.4)	31 (23.1)	
Age at first bacterial infection									
None	752 (64.0)	484 (57.5)	0.006	1094 (61.4)	142 (60.2)	0.096	1159 (61.6)	77 (57.5)	0.057
0 to < 3	116 (9.9)	116 (13.8)		194 (10.9)	38 (16.1)		209 (11.1)	23 (17.2)	
3 to < 6	113 (9.6)	78 (9.3)		173 (9.7)	18 (7.6)		184 (9.8)	7 (5.2)	
6–12	194 (16.5)	164 (19.5)		320 (18.0)	38 (16.1)		331 (17.6)	27 (20.1)	
Total number of infections	2 (0–3)	2 (1–4)	0.002	2 (0–3)	2 (0–4)	0.367	2 (0–4)	2 (0–4)	0.950
Number of viral infections	1 (0–2)	1 (0–3)	0.015	1 (0–2)	1 (0–3)	0.459	1 (0–3)	1 (0–2)	0.584
Number of bacterial infections	0 (0–1)	0 (0–1)	0.005	0 (0–1)	0 (0–1)	0.492	0 (0–1)	0 (0–1)	0.346
Number of infections at age < 3 months	0 (0–1)	0 (0–1)	0.007	0 (0–1)	0 (0–1)	0.039	0 (0–1)	0 (0–1)	0.153
Number of infections at age between 3 and 6 months	0 (0–1)	0 (0–1)	0.119	0 (0–1)	0 (0–1)	0.408	0 (0–1)	0 (0–1)	0.911
Number of infections at age between 6 and 12 months	1 (0–2)	1 (0–2)	0.023	1 (0–2)	1 (0–2)	0.976	1 (0–2)	1 (0–2)	0.457

Values are *n* (%) for categorical variables and median (IQR) for continuous variables

*p* values were calculated using χ^2^ test unless the number of observations in an individual cell was less than 10, then the Fisher’s exact test was utilised. The Wilcoxon rank sum test for continuous variables

AB+, antibody-positive; T1D, type 1 diabetes

**Table 3:** Type of infection in first 12 months of life by antibody and diabetes status

Infection type	AB+ status			Multi-AB+ status			T1D status		
	No AB+ (*n*=1175)	Any AB+ (*n*=842)	*p* value	Not multi-AB+ (*n*=1781)	Multi-AB+ (*n*=236)	*p* value	Did not develop T1D (*n*=1883)	Developed T1D (*n*=134)	*p* value
No infections	338 (28.8)	199 (23.6)	0.015	474 (26.6)	63 (26.7)	0.585	498 (26.4)	39 (29.1)	0.112
Viral only	110 (9.4)	91 (10.8)		182 (10.2)	19 (8.1)		192 (10.2)	9 (6.7)	
Bacterial only	414 (35.2)	285 (33.8)		620 (34.8)	79 (33.5)		661 (35.1)	38 (28.4)	
Viral and bacterial	313 (26.6)	267 (31.7)		505 (28.4)	75 (31.8)		532 (28.3)	48 (35.8)	
Ever had an upper respiratory tract infection									
No	499 (42.5)	320 (38.0)	0.044	729 (40.9)	90 (38.1)	0.411	766 (40.7)	53 (39.6)	0.797
Yes	676 (57.5)	522 (62.0)		1052 (59.1)	146 (61.9)		1117 (59.3)	81 (60.4)	
Ever had gastroenteritis									
No	1006 (85.6)	707 (84.0)	0.307	1510 (84.8)	203 (86.0)	0.619	1598 (84.9)	117 (87.3)	0.424
Yes	169 (14.4)	135 (16.0)		271 (15.2)	33 (14.0)		285 (15.1)	17 (12.7)	
Ever had a urinary tract infection									
No	1143 (97.3)	819 (97.3)	0.991	1729 (97.1)	233 (98.7)	0.144	1828 (97.1)	134 (100.0)	0.048
Yes	32 (2.7)	23 (2.7)		52 (2.9)	3 (1.3)		55 (2.9)	0 (0)	
Ever had a middle ear infection									
No	926 (78.8)	655 (77.8)	0.584	1397 (78.4)	184 (78.0)	0.868	1476 (78.4)	105 (78.4)	0.994
Yes	249 (21.2)	187 (22.2)		384 (21.6)	52 (22.0)		407 (21.6)	29 (21.6)	
Ever had pneumonia									
No	1105 (94.0)	783 (93.0)	0.342	1667 (93.6)	221 (93.6)	0.979	1764 (93.7)	124 (92.5)	0.601
Yes	70 (6.0)	59 (7.0)		114 (6.4)	15 (6.4)		119 (6.3)	10 (7.5)	
Ever had sepsis									
No	1166 (99.2)	833 (98.9)	0.476	1765 (99.1)	234 (99.2)	1.000	1867 (99.2)	132 (98.5)	0.338
Yes	9 (0.8)	9 (1.1)		16 (0.9)	2 (0.8)		16 (0.8)	2 (1.5)	
Ever had any unspecified bacterial infection									
No	1129 (96.1)	786 (93.3)	0.006	1698 (95.3)	217 (91.9)	0.026	1795 (95.3)	120 (89.6)	0.003
Yes	46 (3.9)	56 (6.7)		83 (4.7)	19 (8.1)		88 (4.7)	14 (10.4)	
Ever had any unspecified viral infection									
No	1023 (87.1)	713 (84.7)	0.127	1529 (85.9)	207 (87.7)	0.438	1613 (85.7)	123 (91.8)	0.048
Yes	152 (12.9)	129 (15.3)		252 (14.1)	29 (12.3)		270 (14.3)	11 (8.2)	
Ever had any other viral infection with fever									
No	1075 (91.5)	756 (89.8)	0.192	1616 (90.7)	215 (91.1)	0.855	1704 (90.5)	127 (94.8)	0.098
Yes	100 (8.5)	86 (10.2)		165 (9.3)	21 (8.9)		179 (9.5)	7 (5.2)	
Ever had any antibiotics									
No	779 (66.3)	513 (60.9)	0.013	1143 (64.2)	149 (63.1)	0.754	1209 (64.2)	83 (61.9)	0.597
Yes	396 (33.7)	329 (39.1)		638 (35.8)	87 (36.9)		674 (35.8)	51 (38.1)	

Values are *n* (%)

The definition of ‘other’ or ‘unspecified’ infections, bacterial or viral, means those that are not specified elsewhere in the table

*p* values were calculated using χ^2^ test unless the number of observations in an individual cell was less than 10, then the Fisher’s exact test was utilised

AB+, antibody-positive; T1D, type 1 diabetes

Adjusting for HLA, sex, breastfeeding duration and birth order, those who had seven or more infections during their first year of life were more likely to develop at least one positive type 1 diabetes-related antibody (*p*=0.028, HR 9.166 [95% CI 1.277, 65.81]), when compared with those who did not have any infections (ESM Table 2). Those who had their first viral infection between 6 and 12 months old were less likely to develop at least one positive type 1 diabetes-related antibody (*p*=0.043, HR 0.828 [95% CI 0.690, 0.994]) and multiple antibodies (*p*=0.035, HR 0.664 [95% CI 0.453, 0.972]) (ESM Table 3). Those who ever had an unspecified bacterial infection during their first year of life were more likely to develop at least one positive type 1 diabetes-related antibody (*p*=0.013, HR 1.412 [95% CI 1.075, 1.854]), to develop multiple antibodies (*p*=0.037, HR 1.652 [95% CI 1.030, 2.649]) and to develop clinical type 1 diabetes (*p*=0.011, HR 2.066 [95% CI 1.182, 3.613]) (Table 4; ESM Table 4).

**Table 4:** Univariable and multivariable (adjusted for HLA, gender, breastfeeding duration and birth order) Cox regression results based on time to initial AB+ development, initial multiple AB+ development and type 1 diabetes

	Univariable unadjusted model				Multivariable model			
Outcome	HR	Lower 95% CI	Upper 95% CI	*p* value	HR	Lower 95% CI	Upper 95% CI	*p* value
Any AB+								
Age at first viral infection (reference = those with no infections in first year)								
< 3 months	0.909	0.756	1.093	0.313	0.928	0.771	1.117	0.430
3 to < 6 months	0.952	0.789	1.149	0.610	0.997	0.825	1.205	0.979
6–12 months	0.823	0.686	0.988	0.036	0.828	0.690	0.994	0.043
Number of infections before 3 months (reference = 0)								
1–3	1.088	0.941	1.257	0.255	1.093	0.945	1.265	0.229
4–6	0.377	0.094	1.510	0.168	0.395	0.098	1.584	0.190
7 or more	8.002	1.122	57.09	0.038	9.166	1.277	65.81	0.028
Occurrence of any unspecified bacterial infection	1.379	1.051	1.808	0.020	1.412	1.075	1.854	0.013
Multi-AB+								
Age at first viral infection (reference = those with no infections in first year)								
< 3 months	0.939	0.676	1.305	0.709	0.954	0.684	1.330	0.782
3 to < 6 months	0.709	0.484	1.040	0.079	0.745	0.506	1.097	0.136
6–12 months	0.669	0.457	0.978	0.038	0.664	0.453	0.972	0.035
Number of infections between 6 and 12 months (reference = those with no infections)								
1–3	0.752	0.573	0.988	0.041	0.760	0.578	0.999	0.049
4–6	0.780	0.487	1.251	0.303	0.779	0.485	1.253	0.304
7 or more	1.094	0.508	2.355	0.819	1.076	0.498	2.325	0.852
Occurrence of any unspecified bacterial infection	1.620	1.014	2.589	0.044	1.652	1.030	2.649	0.037
Development of T1D								
Occurrence of any unspecified bacterial infection	2.193	1.261	3.814	0.005	2.066	1.182	3.613	0.011
Occurrence of any unspecified viral infection	0.536	0.289	0.992	0.047	0.525	0.283	0.975	0.041

The definition of ‘other’ or ‘unspecified’ infections, bacterial or viral, means those that are not specified elsewhere in the table

T1D, type 1 diabetes

## Discussion

We used prospectively collected data from the TRIGR study [31] to elucidate the importance of early infections for development of type 1 diabetes.

The majority of participating children had some infections, most commonly classified as viral, recorded during the first year of life; most commonly 1–3 infections. Somewhat surprisingly, among the infants who were not breastfed, a majority had no recorded infection at all, and infections were reported less frequently in children who were not breastfed than among breastfed children, although the opposite was expected [37, 38]. One reason may be that a shorter duration of breastfeeding is associated with lower education and psychosocial problems [39], which may be associated with lower rates of reporting infections.

In univariable analysis (Table 2), those who developed at least one positive type 1 diabetes-related autoantibody were more likely to have experienced both more viral and more bacterial infections during the first 3 months of life compared with those who did not. During their first year of life, these participants also had significantly more infections on average, and more frequent use of antibiotics. Participants who developed multiple antibodies were more likely to have had an unspecified bacterial infection, but were less likely to have had a viral infection, while those who progressed to clinical diabetes were more likely to have had either an unspecified bacterial infection or an unspecified virus infection. We found no significant association with frequent use of antibiotics.

When adjusting for HLA, sex, breastfeeding duration and birth order, those who had their first viral infections at between 6 and 12 months old had a decreased risk of both seroconverting to positivity for multiple autoantibodies and developing type 1 diabetes later in childhood compared with those who did not have any infections in their first year. It should be noted that there is previous evidence for the involvement of viral infection in early life, particularly enterovirus, and also for respiratory infections in particular, in the initiation or progression of islet cell autoimmunity [13]. This evidence cannot be discussed in detail here, but, as an example, virome analysis in the TEDDY study showed that prolonged enterovirus B infection rather than independent, short-duration enterovirus B infections (often asymptomatic) was related to the development of islet cell autoimmunity in young at-risk children [40]. However, our initial analyses did consider whether the occurrence of any respiratory tract infection during the first 12 months was predictive of the development of any autoantibody, multiple autoantibodies and the development of type 1 diabetes. Those who developed at least one positive type 1 diabetes-related antibody more often had an upper respiratory infection, but the association with the occurrence of an unspecified bacterial infection was stronger, as was the association with the use of antibiotics.

Thus, in the current study, certain bacterial infections early in life, especially some less frequent bacterial infections, were associated with a significantly increased risk of both developing autoantibodies later and progressing to clinical type 1 diabetes.

Exposure to infections classified as bacterial in early life appeared to be associated with increased risk of developing both multiple autoantibodies and type 1 diabetes. Such infections may not only influence the immune balance, but also the associated antibiotic treatment may affect the gut microbiota [41], which in turn may influence the immune balance. In a large nationwide birth cohort, antibiotic treatment during the first two years of life was not associated with an increased risk of type 1 diabetes later [42]; however, another nationwide study did find that antibiotic treatment increased the risk of presenting with type 1 diabetes before 10 years old [43].

The strengths of our study are the large sample size, the long follow-up and the prospective documentation of infections in a motivated group of parents of high-risk children who had a family member with type 1 diabetes. However, there are limitations. Infections were documented based on parental report, and reporting quality may have been largely variable between families, with a risk of unreported infections and recall bias. Infections were reported as adverse events. In addition, 78 study centres in 15 countries were involved in the TRIGR study, with the consequent possibility of centre- and country-specific differences in reporting practices. These limitations may be an explanation for the surprising finding that no infections during the first year of life were reported for the majority of children who were never breastfed. Another limitation involves classification of the infections. When antibiotics were used, infections were presumed to be bacterial, but many were not confirmed by specific tests, and we have no record of the specific medical diagnoses; classification was based on information provided by the parents, although that information should usually have been verified by the responsible physician. Another problem is the multiple comparisons. As this study was exploratory, no adjustment was made for multiple comparisons. In general, *p* values < 0.05 were considered significant. However, with stricter requirements for significance, our results tended to favour the importance of bacterial infections over viral infections.

In summary, our results do not support the hypothesis that viral infections in the first year of life initiate the autoimmune process that characterises the development of type 1 diabetes. The finding that those who reported their first viral infection after the age of 6 months had less risk of developing single and multiple autoantibodies compared with those infants who had no infections during in the first year of life implies a protective effect; this fits with the hygiene hypothesis [44] rather than viral infections being a trigger of the autoimmune destruction of the endocrine pancreas. Early life exposure to unspecified bacterial infections appeared to be associated with increased risk of both multiple autoantibodies and type 1 diabetes, possibly via an effect on the intestinal microbiome. In addition, bacterial infections cannot be ruled out as a possible cause of damage to both the exocrine and endocrine pancreas. As this study is post hoc and exploratory by nature, the results require confirmation by other studies.

## Supplementary information

ESM (PDF 820 kb)

## Data Availability

No online repository for TRIGR data is currently available. Information is available upon reasonable request from the TRIGR Data Management Unit at the University of South Florida.

## Abbreviations

GADA: GAD autoantibodies
IA-2A: Tyrosine phosphatase-related insulinoma-associated 2 molecule autoantibodies
IAA: Insulin autoantibodies
TRIGR: Trial to Reduce IDDM in the Genetically at Risk
ZnT8A: Zinc transporter 8 autoantibodies
