# Supplementary material for: Infections in the first year of life and development of beta cell autoimmunity and clinical type 1 diabetes in high-risk individuals: the TRIGR cohort
Source: Diabetologia. 2022 Sep 9;65(12):2098–107. doi: 10.1007/s00125-022-05786-3 (PMC9630400; doi:10.1007/s00125-022-05786-3)
Supplement: Supplementary file 1 — (PDF 820 kb) [file 125_2022_5786_MOESM1_ESM.pdf]

ESM Table 1 Bacterial and Viral Infection timing and number in first 12 months of life by antibody and diabetes status

Infection Number/timing		AB+ Status			Multi-AB+ Status			T1D status		
		No AB+ N=1175	Any AB+ N=842	p-value#	Not Multi-AB+ N=1781	Multi-AB+ N=236	p-value#	Did Not develop T1D N=1883	Developed T1D N=134	p-value#
Number of Infections - % (N)	0	28.8% (338)	23.6% (199)	0.027	26.6% (474)	26.7% (63)	0.665	26.5% (498)	29.1% (39)	0.717
	1-3	47.2% (554)	49.3% (415)		48.5% (863)	44.9% (106)		48.4% (911)	43.3% (58)	
	4-6	18.5% (217)	19.2% (162)		18.5% (330)	20.8% (49)		18.6% (351)	20.9% (28)	
	6 or higher	5.6% (66)	7.8% (66)		6.4% (114)	7.6% (18)		6.5% (123)	6.7% (9)	
Number of Viral Infections - % (N)	0	38.1% (448)	34.4% (290)	0.137	36.8% (656)	34.8% (82)	0.531	36.6% (690)	35.8% (48)	0.480
	1-3	48.3% (568)	49.1% (413)		48.6% (866)	48.7% (115)		48.4% (912)	51.5% (69)	
	4-6	11.5% (135)	13.4% (113)		12.2% (218)	12.7% (30)		12.5% (236)	9.0% (12)	
	6 or higher	2.0% (24)	3.1% (26)		2.3% (41)	3.8% (9)		2.4% (45)	3.7% (5)	
Number of Bacterial Infections - % (N)	0	64.0% (752)	57.5% (484)	0.015	61.4% (1094)	60.2% (142)	0.940	61.6% (1159)	57.5% (77)	0.425
	1-3	32.3% (380)	39.1% (329)		35.1% (625)	35.6% (84)		35.0% (659)	37.3% (50)	
	4-6	3.2% (38)	3.2% (27)		3.1% (56)	3.8% (9)		3.1% (58)	5.2% (7)	
	6 or higher	0.4% (5)	0.2% (2)		0.3% (6)	0.4% (1)		0.4% (7)	0% (0)	
Number of Infections <3 mos of age - % (N)	0	72.9% (856)	67.5% (568)	0.007	71.3% (1270)	65.3% (154)	0.147	71.0% (1336)	65.7% (88)	0.354
	1-3	26.5% (311)	32.2% (271)		28.1% (500)	34.8% (82)		28.5% (536)	34.3% (46)	
	4-6	0.7% (8)	0.2% (2)		0.6% (10)	0% (0)		0.5% (10)	0% (0)	
	6 or higher	0% (0)	0.1% (1)		0.1% (6)	0% (0)		0.1% (1)	0% (0)	
Number of Infections between 3 and 6 mos of age - % (N)	0	65.3% (767)	62.6% (527)	0.633	64.3% (1145)	63.1% (149)	0.493	64.2% (1208)	64.2% (86)	0.846
	1-3	33.5% (394)	36.1% (304)		34.5% (615)	35.2% (83)		34.6% (652)	34.3% (46)	
	4-6	1.0% (12)	1.2% (10)		1.1% (19)	1.3% (3)		1.1% (20)	1.5% (2)	
	6 or higher	0.2% (2)	0.1% (1)		0.1% (2)	0.4% (1)		0.2% (3)	0% (0)	
Number of Infections	0	42.0% (494)	37.3% (314)	0.091	39.9% (711)	41.1% (97)	0.744	39.9% (751)	42.5% (57)	0.942

between 6 and 12 mos of age - % (N)	1-3	47.7% (561)	51.3% (432)		49.5% (882)	47.0% (111)		49.4% (930)	47.0% (63)	
	4-6	8.5% (100)	8.7% (73)		8.5% (152)	8.9% (21)		8.6% (162)	8.2% (11)	
	6 or higher	1.7% (20)	2.7% (23)		2.0% (36)	3.0% (7)		2.1% (40)	2.2% (3)	

# p-values based on Chi- Square test or Fisher's Exact test (based on cell size)

ESM Table 2 Univariable and Multivariable (adjusted for HLA, Gender, Breastfeeding Duration and Birth Order) Cox regression results based on time to initial AB+ development. The definition other or unspecified infections, bacterial or viral, means previously not specified as eg pneumonia, sepsis etc

		Univariate Model Unadjusted Model				Multivariate Model Adjusted by HLA, Gender, Breastfeeding Duration and Birth Order			
**Parameter**	**Level**	**p-value**	**Hazard Ratio**	**Lower 95% CL**	**Upper 95% CL**	**p-value**	**Hazard Ratio**	**Lower 95% CL**	**Upper 95% CL**
Age at First Infection (Categorical) (Ref = None reported)	0-<3 mos	0.7065	0.966	0.804	1.159	0.8889	0.987	0.820	1.187
	3-<6 mos	0.1426	0.859	0.702	1.052	0.2953	0.896	0.730	1.100
	6-12 mos	0.0882	0.842	0.690	1.026	0.1087	0.850	0.696	1.037
Age at First Viral Infection (Categorical) (Ref = None reported)	0-<3 mos	0.3126	0.909	0.756	1.093	0.4275	0.928	0.771	1.116
	3-<6 mos	0.6101	0.952	0.789	1.149	0.9521	0.994	0.823	1.201
	6-12 mos	0.0362	0.823	0.686	0.988	0.0425	0.828	0.689	0.994
Age at First Bacterial Infection (Categorical) (Ref = None reported)	0-<3 mos	0.0778	1.200	0.980	1.470	0.0683	1.210	0.986	1.484
	3-<6 mos	0.5088	0.923	0.726	1.172	0.6438	0.945	0.743	1.202
	6-12 mos	0.8429	1.018	0.853	1.215	0.7048	1.035	0.866	1.237
Number of Infections (Categorical) (Ref = 0)	1-3	0.2287	0.901	0.761	1.067	0.3085	0.915	0.772	1.085
	4-6	0.0807	0.831	0.675	1.023	0.1776	0.866	0.702	1.068
	6 or higher	0.7118	1.054	0.798	1.392	0.5550	1.089	0.821	1.445
Number of Infections (Continuous)		0.9440	0.999	0.973	1.026	0.8046	1.003	0.977	1.031
Number of Viral Infections (Categorical) (Ref = 0)	1-3	0.0546	0.863	0.743	1.003	0.0904	0.878	0.755	1.021
	4-6	0.5689	0.939	0.755	1.167	0.8159	0.974	0.783	1.213
	6 or higher	0.3738	1.200	0.803	1.792	0.2917	1.244	0.829	1.866
Number of Viral Infections (Continuous)		0.9384	1.001	0.968	1.036	0.6572	1.008	0.974	1.043
Number of Bacterial Infections (Categorical) (Ref = 0)	1-3	0.3382	1.071	0.931	1.232	0.2434	1.088	0.944	1.254
	4-6	0.4702	0.867	0.588	1.277	0.5112	0.877	0.594	1.296
	6 or higher	0.3412	0.509	0.127	2.043	0.2869	0.469	0.117	1.889
Number of Bacterial Infections (Continuous)		0.8044	0.993	0.943	1.047	0.8501	0.995	0.944	1.049
Infection Types (Categorical) (Ref = No Infections)	Bacterial Inf Only	0.0646	0.843	0.703	1.010	0.1068	0.861	0.718	1.033
	Viral Inf Only	0.8625	0.978	0.763	1.254	0.9558	0.993	0.772	1.277
	Viral and Bacterial	0.4444	0.931	0.775	1.118	0.6747	0.961	0.798	1.158
	1-3	0.2553	1.088	0.941	1.257	0.2298	1.093	0.945	1.265

		Univariate Model Unadjusted Model				Multivariate Model Adjusted by HLA, Gender, Breastfeeding Duration and Birth Order			
Parameter	Level	p-value	Hazard Ratio	Lower 95% CL	Upper 95% CL	p-value	Hazard Ratio	Lower 95% CL	Upper 95% CL
Number of Infections before 3 mos (Categorical) (Ref = 0)	4-6	0.1680	0.377	0.094	1.510	0.1827	0.389	0.097	1.561
	6 or higher	0.0380	8.002	1.122	57.09	0.0278	9.146	1.273	65.71
Number of Infections before 3 mos (Continuous)		0.3255	1.042	0.960	1.132	0.2611	1.049	0.965	1.140
Number of Infections between 3 and 6 mos (Categorical) (Ref = 0)	1-3	0.7943	0.981	0.852	1.130	0.9093	1.008	0.874	1.163
	4-6	0.9009	0.961	0.514	1.797	0.8623	1.057	0.562	1.988
	6 or higher	0.7918	0.768	0.108	5.460	0.7708	0.747	0.105	5.325
Number of Infections between 3 and 6 mos (Continuous)		0.8145	1.009	0.939	1.084	0.4878	1.026	0.954	1.104
Number of Infections between 6 and 12 mos (Categorical) (Ref = 0)	1-3	0.1138	0.889	0.769	1.028	0.1486	0.898	0.776	1.039
	4-6	0.0559	0.780	0.605	1.006	0.0808	0.796	0.616	1.028
	6 or higher	0.2905	1.256	0.823	1.918	0.2424	1.290	0.842	1.975
Number of Infections between 6 and 12 mos (Continuous)		0.5072	0.987	0.949	1.026	0.6112	0.990	0.952	1.030
Occurrence of ANY Upper Respiratory Infection		0.3055	0.930	0.809	1.069	0.5675	0.960	0.834	1.105
Occurrence of ANY Gastroenteritis		0.5964	0.951	0.791	1.144	0.9069	0.989	0.822	1.190
Occurrence of ANY Urinary Tract Infection		0.8988	0.973	0.643	1.473	0.8991	0.974	0.643	1.474
Occurrence of ANY Middle Ear Infection		0.1497	0.887	0.754	1.044	0.1743	0.892	0.757	1.052
Occurrence of ANY Pneumonia		0.7329	1.047	0.804	1.364	0.6911	1.056	0.807	1.381
Occurrence of ANY Sepsis		0.7627	1.106	0.574	2.134	0.7266	1.125	0.582	2.172
Occurrence of ANY Other Bacterial Infection		0.0203	1.379	1.051	1.808	0.0135	1.410	1.073	1.852
Occurrence of ANY Other Viral Infection		0.8093	1.023	0.848	1.234	0.8010	1.025	0.849	1.237

		Univariate Model Unadjusted Model				Multivariate Model Adjusted by HLA, Gender, Breastfeeding Duration and Birth Order			
**Parameter**	**Level**	**p-value**	**Hazard Ratio**	**Lower 95% CL**	**Upper 95% CL**	**p-value**	**Hazard Ratio**	**Lower 95% CL**	**Upper 95% CL**
Occurrence of ANY Other Viral Infection with Fever		0.9893	0.998	0.799	1.248	0.9567	1.006	0.805	1.258
Occurrence of ANY Antibiotic Use		0.8878	1.010	0.879	1.160	0.7466	1.023	0.889	1.178

ESM Table 3 Univariable and Multivariable (adjusted for HLA, Gender, Breastfeeding Duration and Birth Order) Cox regression results based on time to initial multiple-AB+ development. The definition other or unspecified infections, bacterial or viral, means previously not specified as eg pneumonia, sepsis etc

		Univariate Model Unadjusted Model				Multivariate Model Adjusted by HLA, Gender, Breastfeeding Duration and Birth Order			
**Parameter**	**Level**	**p-value**	**Hazard Ratio**	**Lower 95% CL**	**Upper 95% CL**	**p-value**	**Hazard Ratio**	**Lower 95% CL**	**Upper 95% CL**
Age at First Infection (Categorical) (Ref = None reported)	0-<3 mos	0.7087	0.939	0.676	1.305	0.7400	0.945	0.678	1.318
	3-<6 mos	0.0787	0.709	0.484	1.040	0.1108	0.730	0.496	1.075
	6-12 mos	0.0381	0.669	0.457	0.978	0.0324	0.660	0.451	0.966
Age at First Viral Infection (Categorical) (Ref = None reported)	0-<3 mos	0.6523	0.923	0.652	1.307	0.7531	0.946	0.667	1.341
	3-<6 mos	0.9999	1.000	0.705	1.419	0.8285	1.040	0.732	1.477
	6-12 mos	0.2283	0.807	0.569	1.144	0.2079	0.798	0.562	1.133
Age at First Bacterial Infection (Categorical) (Ref = None reported)	0-<3 mos	0.1160	1.332	0.932	1.906	0.1467	1.308	0.910	1.880
	3-<6 mos	0.1929	0.722	0.442	1.179	0.1939	0.722	0.441	1.181
	6-12 mos	0.2500	0.810	0.567	1.159	0.2591	0.813	0.567	1.165
Number of Infections (Categorical) (Ref = 0)	1-3	0.0769	0.755	0.553	1.031	0.0761	0.753	0.550	1.030
	4-6	0.3450	0.835	0.575	1.214	0.4303	0.859	0.589	1.253
	6 or higher	0.6401	0.882	0.523	1.490	0.6309	0.878	0.516	1.493
Number of Infections (Continuous)		0.7214	1.009	0.961	1.060	0.6990	1.010	0.961	1.061
Number of Viral Infections (Categorical) (Ref = 0)	1-3	0.4073	0.887	0.668	1.178	0.4490	0.896	0.675	1.190
	4-6	0.5259	0.873	0.575	1.327	0.6581	0.909	0.597	1.386
	6 or higher	0.3697	1.370	0.688	2.728	0.3891	1.357	0.677	2.718
Number of Viral Infections (Continuous)		0.9423	1.002	0.940	1.069	0.8191	1.007	0.945	1.074
Number of Bacterial Infections (Categorical) (Ref = 0)	1-3	0.5848	0.928	0.708	1.215	0.5798	0.926	0.705	1.216
	4-6	0.9307	1.030	0.525	2.021	0.9857	1.006	0.510	1.984
	6 or higher	0.9577	0.948	0.133	6.779	0.7921	0.767	0.106	5.524
Number of Bacterial Infections (Continuous)		0.5544	1.028	0.937	1.128	0.6898	1.019	0.929	1.118
Infection Types (Categorical) (Ref = No Infections)	Bacterial Inf Only	0.1228	0.770	0.553	1.073	0.1391	0.778	0.558	1.085
	Viral Inf Only	0.1144	0.662	0.396	1.105	0.1000	0.647	0.386	1.087
	Viral and Bacterial	0.3409	0.850	0.608	1.188	0.3576	0.853	0.607	1.197
	1-3	0.1522	1.216	0.930	1.590	0.1493	1.219	0.931	1.597

		Univariate Model Unadjusted Model				Multivariate Model Adjusted by HLA, Gender, Breastfeeding Duration and Birth Order			
**Parameter**	**Level**	**p-value**	**Hazard Ratio**	**Lower 95% CL**	**Upper 95% CL**	**p-value**	**Hazard Ratio**	**Lower 95% CL**	**Upper 95% CL**
Number of Infections before 3 mos (Categorical) (Ref = 0)	4-6	0.9723	0.000	0.000	2E287	0.9727	0.000	0.000	7E291
	6 or higher	0.9912	0.000	0.000	.	0.9915	0.000	0.000	.
Number of Infections before 3 mos (Continuous)		0.2214	1.094	0.947	1.263	0.1927	1.102	0.952	1.275
Number of Infections between 3 and 6 mos (Categorical) (Ref = 0)	1-3	0.6138	0.933	0.713	1.221	0.7240	0.952	0.725	1.250
	4-6	0.9912	1.006	0.321	3.157	0.9519	1.036	0.327	3.279
	6 or higher	0.2594	3.101	0.434	22.17	0.3469	2.578	0.358	18.54
Number of Infections between 3 and 6 mos (Continuous)		0.4220	1.055	0.926	1.201	0.3526	1.064	0.933	1.213
Number of Infections between 6 and 12 mos (Categorical) (Ref = 0)	1-3	0.0407	0.752	0.573	0.988	0.0379	0.749	0.570	0.984
	4-6	0.3029	0.780	0.487	1.251	0.2881	0.773	0.481	1.243
	6 or higher	0.8192	1.094	0.508	2.355	0.8416	1.082	0.501	2.337
Number of Infections between 6 and 12 mos (Continuous)		0.6493	0.983	0.913	1.058	0.6143	0.981	0.911	1.057
Occurrence of ANY Upper Respiratory Infection		0.6316	0.938	0.721	1.220	0.8162	0.969	0.743	1.263
Occurrence of ANY Gastroenteritis		0.2818	0.817	0.566	1.180	0.5163	0.885	0.611	1.281
Occurrence of ANY Urinary Tract Infection		0.1637	0.445	0.143	1.390	0.1716	0.452	0.145	1.412
Occurrence of ANY Middle Ear Infection		0.4826	0.896	0.658	1.218	0.4402	0.885	0.649	1.207
Occurrence of ANY Pneumonia		0.8284	0.944	0.559	1.592	0.6743	0.893	0.525	1.517
Occurrence of ANY Sepsis		0.8292	0.858	0.213	3.451	0.8671	0.888	0.220	3.577
Occurrence of ANY Other Bacterial Infection		0.0438	1.620	1.014	2.589	0.0379	1.649	1.028	2.644
Occurrence of ANY Other Viral Infection		0.2078	0.779	0.528	1.149	0.1933	0.772	0.523	1.140

		Univariate Model Unadjusted Model				Multivariate Model Adjusted by HLA, Gender, Breastfeeding Duration and Birth Order			
Parameter	Level	p-value	Hazard Ratio	Lower 95% CL	Upper 95% CL	p-value	Hazard Ratio	Lower 95% CL	Upper 95% CL
Occurrence of ANY Other Viral Infection with Fever		0.4654	0.846	0.541	1.325	0.5202	0.863	0.551	1.352
Occurrence of ANY Antibiotic Use		0.5543	0.923	0.709	1.203	0.5160	0.915	0.700	1.196

ESM Table 4 Univariable and Multivariable (adjusted for HLA, Gender, Breastfeeding Duration and Birth Order) Cox regression results based on time to type-1 diabetes. The definition other or unspecified infections, bacterial or viral, means previously not specified as eg pneumonia, sepsis etc

		Univariate Model Unadjusted Model				Multivariate Model Adjusted by HLA, Gender, Breastfeeding Duration and Birth Order			
Parameter	Level	p-value	Hazard Ratio	Lower 95% CL	Upper 95% CL	p-value	Hazard Ratio	Lower 95% CL	Upper 95% CL
Age at First Infection (Categorical) (Ref = None reported)	0-<3 mos	0.9152	0.977	0.638	1.497	0.9856	0.996	0.645	1.538
	3-<6 mos	0.0898	0.636	0.377	1.073	0.1159	0.654	0.385	1.110
	6-12 mos	0.2713	0.759	0.465	1.240	0.2639	0.755	0.461	1.236
Age at First Viral Infection (Categorical) (Ref = None reported)	0-<3 mos	0.7865	0.938	0.588	1.495	0.9277	0.978	0.611	1.566
	3-<6 mos	0.9645	0.989	0.617	1.586	0.8946	1.033	0.642	1.660
	6-12 mos	0.8871	0.968	0.616	1.521	0.9568	0.988	0.628	1.553
Age at First Bacterial Infection (Categorical) (Ref = None reported)	0-<3 mos	0.0576	1.570	0.986	2.502	0.0627	1.565	0.976	2.510
	3-<6 mos	0.1443	0.562	0.259	1.218	0.1661	0.578	0.266	1.256
	6-12 mos	0.4465	1.186	0.765	1.838	0.4191	1.200	0.771	1.869
Number of Infections (Categorical) (Ref = 0)	1-3	0.1923	0.763	0.509	1.146	0.2069	0.768	0.510	1.157
	4-6	0.7386	0.921	0.566	1.496	0.8630	0.958	0.585	1.568
	6 or higher	0.6167	0.831	0.402	1.716	0.6295	0.834	0.400	1.742
Number of Infections (Continuous)		0.8001	0.991	0.926	1.061	0.8792	0.995	0.928	1.066
Number of Viral Infections (Categorical) (Ref = 0)	1-3	0.9190	1.019	0.705	1.474	0.8043	1.048	0.724	1.516
	4-6	0.2141	0.670	0.356	1.261	0.2929	0.710	0.376	1.343
	6 or higher	0.4742	1.400	0.557	3.518	0.4449	1.438	0.567	3.648

		Univariate Model Unadjusted Model				Multivariate Model Adjusted by HLA, Gender, Breastfeeding Duration and Birth Order			
**Parameter**	**Level**	**p-value**	**Hazard Ratio**	**Lower 95% CL**	**Upper 95% CL**	**p-value**	**Hazard Ratio**	**Lower 95% CL**	**Upper 95% CL**
Number of Viral Infections (Continuous)		0.4299	0.964	0.880	1.056	0.5545	0.973	0.888	1.066
Number of Bacterial Infections (Categorical) (Ref = 0)	1-3	0.5729	1.108	0.776	1.582	0.5255	1.124	0.784	1.612
	4-6	0.2050	1.650	0.761	3.577	0.2176	1.635	0.748	3.573
	6 or higher	0.9745	0.000	0.000	2E287	0.9740	0.000	0.000	3E289
Number of Bacterial Infections (Continuous)		0.4612	1.047	0.927	1.183	0.5479	1.038	0.919	1.172
Infection Types (Categorical) (Ref = No Infections)	Bacterial Inf Only	0.0918	0.681	0.435	1.064	0.1096	0.693	0.442	1.086
	Viral Inf Only	0.1348	0.575	0.279	1.187	0.1138	0.554	0.266	1.152
	Viral and Bacterial	0.8230	1.049	0.688	1.602	0.7167	1.083	0.704	1.666
Number of Infections before 3 mos (Categorical) (Ref = 0)	1-3	0.2428	1.237	0.866	1.767	0.2123	1.257	0.877	1.802
	4-6	0.9798	0.000	0.000	.	0.9801	0.000	0.000	.
	6 or higher	0.9939	0.000	0.000	.	0.9943	0.000	0.000	.
Number of Infections before 3 mos (Continuous)		0.3293	1.100	0.908	1.334	0.2749	1.115	0.917	1.355
Number of Infections between 3 and 6 mos (Categorical) (Ref = 0)	1-3	0.7090	0.934	0.653	1.336	0.8110	0.957	0.665	1.376
	4-6	0.7892	1.211	0.298	4.923	0.6881	1.336	0.324	5.508
	6 or higher	0.9765	0.000	0.000	9E285	0.9766	0.000	0.000	2E292
Number of Infections between 3 and 6 mos (Continuous)		0.9628	1.004	0.835	1.207	0.8275	1.021	0.848	1.229
Number of Infections between 6 and 12 mos (Categorical) (Ref = 0)	1-3	0.3330	0.838	0.585	1.199	0.3443	0.841	0.586	1.205
	4-6	0.5738	0.831	0.436	1.585	0.6161	0.847	0.442	1.623
	6 or higher	0.9260	0.946	0.296	3.023	0.9617	0.972	0.303	3.117
Number of Infections between 6 and 12 mos (Continuous)		0.4074	0.957	0.863	1.062	0.4135	0.957	0.862	1.063
Occurrence of ANY Upper Respiratory Infection		0.8999	0.978	0.692	1.383	0.8616	1.032	0.727	1.465
Occurrence of ANY Gastroenteritis		0.3809	0.797	0.479	1.325	0.5979	0.871	0.522	1.454
Occurrence of ANY Urinary Tract Infection		0.9702	0.000	0.000	3E291	0.9706	0.000	0.000	7E294

Parameter	Level	Univariate Model Unadjusted Model				Multivariate Model Adjusted by HLA, Gender, Breastfeeding Duration and Birth Order			
		p-value	Hazard Ratio	Lower 95% CL	Upper 95% CL	p-value	Hazard Ratio	Lower 95% CL	Upper 95% CL
Occurrence of ANY Middle Ear Infection		0.8357	0.957	0.634	1.445	0.8815	0.969	0.640	1.468
Occurrence of ANY Pneumonia		0.5329	1.228	0.644	2.339	0.5166	1.241	0.646	2.386
Occurrence of ANY Sepsis		0.4506	1.712	0.424	6.917	0.4668	1.682	0.415	6.818
Occurrence of ANY Other Bacterial Infection		0.0054	2.193	1.261	3.814	0.0107	2.069	1.184	3.616
Occurrence of ANY Other Viral Infection		0.0472	0.536	0.289	0.992	0.0355	0.515	0.277	0.956
Occurrence of ANY Other Viral Infection with Fever		0.0928	0.521	0.243	1.115	0.0951	0.522	0.244	1.120
Occurrence of ANY Antibiotic Use		0.7118	1.068	0.753	1.514	0.6264	1.092	0.766	1.556

ESM Figure 1 Kaplan-Meier curve of time from birth to development of any positive antibody by A) Age at first viral infection, B) Number of infections before 3 mos, C) Occurrence of any unspecified bacterial infection

A.

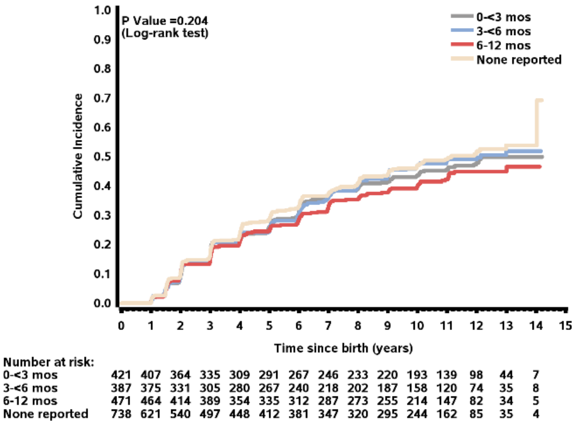

B.

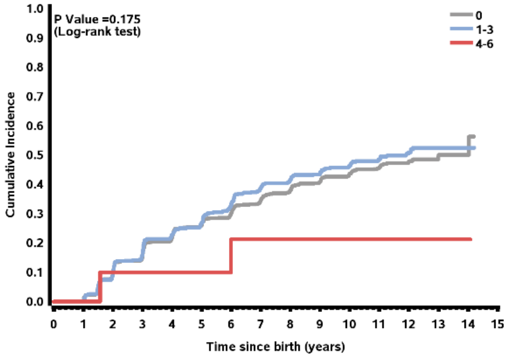

Number at risk:

0	1424	1291	1140	1055	961	899	836	766	713	661	558	386	215	95	16
1-3	582	565	499	462	421	398	357	325	308	289	245	178	120	50	7
4-6	10	10	9	9	9	8	7	7	7	7	6	4	4	3	1

C.

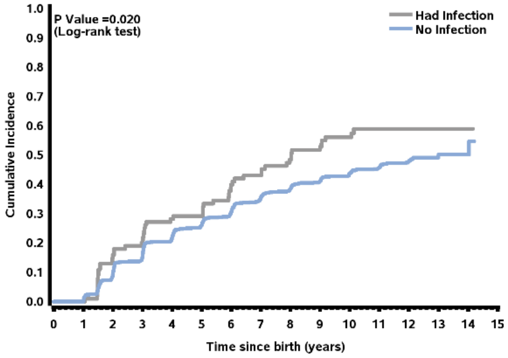

Number at risk:

Had Infection	102	100	84	78	70	67	56	53	48	43	33	21	12	6	2
No Infection	1915	1767	1565	1448	1321	1238	1144	1045	980	914	776	547	327	142	22

ESM Figure 2 Kaplan-Meier curve of time from birth to development of multi-positive antibody by A) Age at first viral infection, B) Number of infections between 6 and 12 mos, C) Occurrence of any unspecified bacterial infection

A.

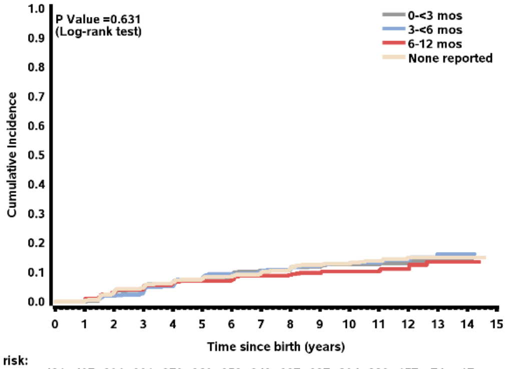

Number at risk:															
0-<3 mos	421	407	391	381	370	360	353	343	337	327	294	223	157	74	17
3-<6 mos	387	375	358	347	339	328	315	308	301	292	268	198	129	70	15
6-12 mos	471	464	446	435	417	411	402	390	385	370	326	234	132	59	12
None reported	738	624	585	567	550	530	519	498	480	463	409	285	166	76	13

B.

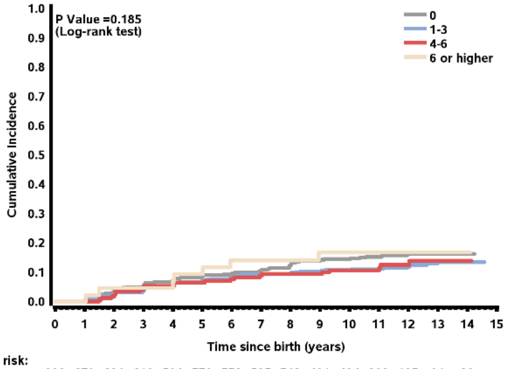

Number at risk:															
0	808	679	631	610	591	570	558	535	512	491	434	303	185	91	20
1-3	993	976	941	917	886	865	843	818	808	781	698	513	317	143	30
4-6	173	172	167	162	159	156	154	152	149	149	135	103	68	40	6
6 or higher	43	43	41	41	40	38	34	34	34	31	30	21	14	5	1

C.

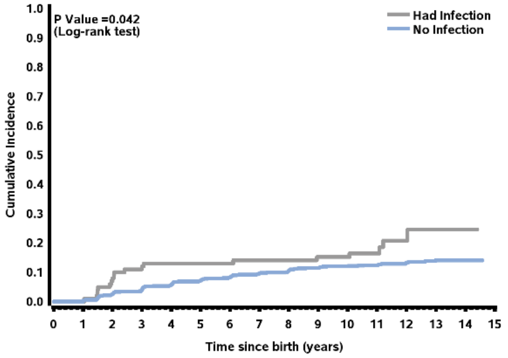

Number at risk:															
Had Infection	102	100	93	87	85	82	81	80	80	78	69	45	21	13	5
No Infection	1915	1770	1687	1643	1591	1547	1508	1459	1423	1374	1228	895	563	266	52

ESM Figure 3 Kaplan-Meier curve of time from birth to development of type-1 diabetes by A) Occurrence of any unspecified bacterial infection, B) Occurrence of any unspecified viral infection

A.

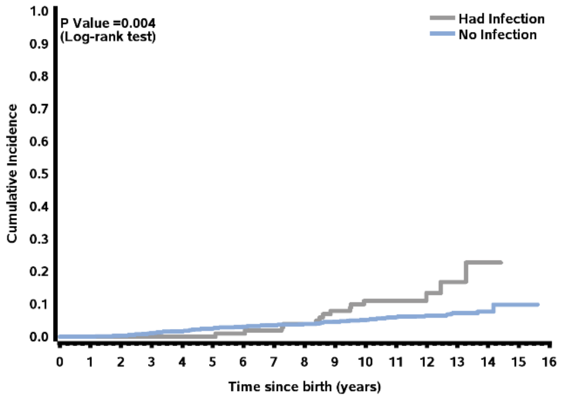

Number at risk:																
Had Infection	102	102	102	102	102	102	101	99	96	92	83	59	35	21	6	
No Infection	1915	1909	1896	1874	1849	1819	1790	1771	1759	1729	1632	1260	837	418	105	3

B.

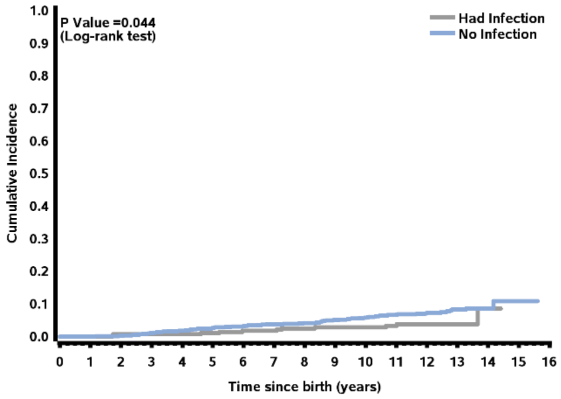

Number at risk:																
Had Infection	281	281	278	278	277	275	272	270	267	263	248	198	137	62	11	
No Infection	1736	1730	1720	1698	1674	1646	1619	1600	1588	1558	1467	1121	735	377	100	3

LIST OF TRIGR INVESTIGATORS

Administration/ Country	Study center	Last name	First name	Position
**Data Safety Monitoring Board**		Mandrup-Poulsen,	Thomas	Chair University of Copenhagen, Copenhagen, Denmark
		Arjas	Elias	Member, University of Helsinki, Helsinki, Finland
		Läärä	Esa	Member, University of Oulu, Oulu, Finland
		Lernmark	Åke	Member, University of Lund, Malmö, Sweden
		Schmidt	Barbara	Member, University of Pennsylvania, Philadelphia, PA, USA
		Krischer	Jeffrey P.	Observer, University of South Florida. Tampa FL, USA
**International Coordinating Center (ICC), University of Helsinki, Helsinki, Finland**		Åkerblom	Hans K. † *	PI of the Study until 30.6.08, Deputy PI from 1.7.2008
		Hyytinen	Mila	European Study Monitor
		Knip	Mikael	Deputy PI until 30.6.2008, PI of the Study from 1.7.2008, National Investigator
		Koski	Katriina	European Study Monitor
		Koski	Matti	IT Specialist
		Pajakkala	Eeva	European Study Monitor
		Salonen	Marja	Study Coordinator
**Data Management Unit (DMU), University of South Florida, Tampa, Florida, USA**		Cuthbertson	David	Biostatistician
		Krischer	Jeffrey P.	PI of the DMU
		Shanker	Linda	Coordinator

LIST OF TRIGR INVESTIGATORS

Administration/ Country	Study center	Last name	First name	Position
**Canadian Coordinating Center, University of Western Ontario, London, and University of Ottawa, Ontario**		Bradley	Brenda	National Coordinator
		Dosch	Hans-Michael	Co-Investigator for Canada
		Dupré	John † *	Co-PI for North America and National Investigator until 08/12/2015, Executive Committee
		Fraser	William	Co-Investigator for Canada Executive Committee
		Lawson	Margaret	Co-Investigator for Canada Executive Committee
		Mahon	Jeffrey L.	Co-PI for North America and National Investigator after 08/12/2015, Executive Committee
		Sermer	Mathew	Co-Investigator for Canada, Executive Committee
		Taback	Shayne P.	Co-Investigator for Canada, Executive Committee
**USA Coordinating Center, University of Pittsburgh, Pennsylvania and University of Washington, Seattle, Washington**		Becker	Dorothy	Co-PI for North America, National Investigator, Executive Committee
		Franciscus	Margaret	National Coordinator
		Nucci	Anita	National Coordinator, Nutrition Coordinator of North America
		Palmer	Jerry	Executive Committee

LIST OF TRIGR INVESTIGATORS

Administration/ Country	Study center	Last name	First name	Position
**Nutritional Epidemiology Unit, National Institute for Health and Welfare, Helsinki, Finland**		Virtanen	Suvi M.	Head of Nutritional Epidemiology Unit
**Australia**	**AUS01 – Westmead, New South Wales - Children's Hospital**	Catteau<br>Howard	Jacki<br>Neville	National Coordinator<br>National Investigator
	**AUS02 – Newcastle, New South Wales - John Hunter Children's Hospital**	Crock	Patricia	Local Investigator
	**AUS03 – Sydney, New South Wales - Sydney Children's Hospital**	Craig	Maria	Local Investigator
**Canada**	**CAN01 – London, Ontario - St. Joseph's Health Care Centre**	Clarson<br>Bere	Cheril L.<br>Lynda	Local Investigator<br>Coordinator
	**CAN02 – Vancouver, British Columbia - Children's and Women's Health Centre of British Columbia**	Thompson<br>Metzger<br>Marshall<br>Kwan	David<br>Daniel<br>Colleen<br>Jennifer	Local Investigator<br>Local Investigator<br>Coordinator (In Transition)<br>Coordinator (In Transition)
	**CAN03 – Calgary, Alberta - Alberta Children's Hospital**	Stephure<br>Pacaud<br>Schwarz	David K.<br>Daniele<br>Wendy	Local Investigator<br>Co-Investigator<br>Coordinator

LIST OF TRIGR INVESTIGATORS

Administration/ Country	Study center	Last name	First name	Position
	**CAN04 – Edmonton, Alberta - Walter MacKenzie Health Sciences**	Girgis<br>Thompson	Rose<br>Marilyn	Local Investigator<br>Coordinator
	**CAN05 – Winnipeg, Manitoba - Health Sciences Centre**	Taback<br>Catte	Shayne P<br>Daniel	Local Investigator<br>Coordinator
	**CAN06 – Ottawa, Ontario - Children's Hospital of Eastern Ontario and The Ottawa Hospital**	Lawson<br>Bradley	Margaret L<br>Brenda	Local Investigator<br>Coordinator
	**CAN07 – Toronto, Ontario - Mount Sinai Hospital/Hospital for Sick Children**	Daneman<br>Sermer<br>Martin	Denis<br>Mathew<br>Mary-Jean	Local Investigator<br>Co-Investigator<br>Coordinator
	**CAN08 – Quebec, Quebec - CHUQ**	Morin<br>Frenette<br>Ferland	Valérie<br>Line<br>Suzanne	Local Investigator<br>Co-Investigator<br>Coordinator
	**CAN09 - Saint John, New Brunswick – Regional Hospital**	Sanderson<br>Heath	Susan<br>Kathy	Local Investigator<br>Coordinator
	**CAN10 – Montreal, Quebec - L' Hôpital Sainte-Justine**	Huot<br>Gonthier<br>Thibeault	Céline	Local Investigator<br>Co-Investigator<br>Coordinator
	**CAN11 – Montreal, Quebec - Children's**	Legault<br>Laforte	Laurent<br>Diane	Local Investigator<br>Coordinator

LIST OF TRIGR INVESTIGATORS

Administration/ Country	Study center	Last name	First name	Position
	**Hospital**			
	**CAN12 – Halifax, Nova Scotia - IWK Health Centre/Dalhousie**	Cummings<br>Scott	Elizabeth A<br>Karen	Local Investigator<br>Coordinator
	**CAN13 - St. John's, Newfoundland and Labrador - Janeway Child Health Center**	Bridger<br>Crummell	Tracey<br>Cheryl	Local Investigator<br>Coordinator
	**CAN14 – Kingston, Ontario - Kingston General Hospital/ Queen's University**	Houlden<br>Breen	Robyn<br>Adriana	Local Investigator<br>Coordinator
	**CAN15 – Regina, Saskatchewan - Regina Qu'Appelle**	Carson<br>Kelly	George<br>Sheila	Local Investigator<br>Coordinator
	**CAN16 – Saskatoon, Saskatchewan - Royal University Hospital**	Sankaran<br>Penner	Koravangattu<br>Marie	Local Investigator<br>Coordinator
	**CAN17 – Peterborough, Ontario - Peterborough Regional Health Centre**	White<br>King	Richard A<br>Nancy	Local Investigator<br>Coordinator
	**CAN18 – Victoria, British Columbia - Vancouver Island Health Research Centre**	Popkin<br>Robson	James<br>Laurie	Local Investigator<br>Coordinator

LIST OF TRIGR INVESTIGATORS

Administration/ Country	Study center	Last name	First name	Position
**Czech Republic**	**CZE01 - Prague - Faculty Hospital Kralovske Vinohrady**	Al Taji	Eva	National Investigator/Coordinator
		Mendlova	Pavla	National Coordinator
		Romanova	Martina	Co-Investigator
		Vavrinec	Jan † *	National Investigator
		Vosahlo	Jan	Co-Investigator
	**CZE02 - Brno - Hospital Milosrdnych Bratri**	Brazdova	Ludmila	Local Investigator
	**CZE03- Olomouc - Faculty Hospital Olomouc**	Venhacova	Jitrenka	Local Investigator
		Venhacova	Petra	Co-Investigator
	**CZE04 - Usti nad Labem - Hospital of Masryk**	Cipra	Adam	Local Investigator
	**CZE05 - Ceske Budejovice - Hospital Ceske Budejovice**	Tomsikova	Zdenka	Local Investigator
	**CZE06 - Plzen - Faculty Hospital Plzen**	Paterová	Petra	Local Investigator
	**CZE07 - Zlin - Hospital of Bata**	Gogelova	Pavla	Local Investigator
**Estonia**	**EST01 - Tallinn - Tallinn Children’s Hospital**	Einberg	Ülle	Co-Investigator
		Riikjärv	Mall-Anne	Local Investigator
	**EST02 - Tartu - Tartu**	Ormisson	Anne	National Investigator

LIST OF TRIGR INVESTIGATORS

Administration/ Country	Study center	Last name	First name	Position
	**University Children's Hospital**	Tillmann	Vallo	Co-Investigator

LIST OF TRIGR INVESTIGATORS

Administration/ Country	Study center	Last name	First name	Position
Finland	FIN01 - Helsinki – Children's Hospital, University of Helsinki	Johansson	Susanne	National Coordinator
		Kleemola	Päivi	National Coordinator
		Parkkola	Anna	Local Investigator
	FIN02 - Helsinki - Department of Obstetrics and Gynecology, University of Helsinki	Järvenpää	Anna-Liisa	Local Investigator
	FIN03 - Espoo - Jorvi Hospital	Hämäläinen	Anu-Maaria	Local Investigator
		Kiiveri	Sanne	Local Investigator
	FIN04 - Kotka - Kymenlaakso Central Hospital	Salonen	Maria	Local Investigator
		Tenhola	Sirpa	Local Investigator
	FIN05 - Lahti - Paijat-Hame Central Hospital	Salonen	Pia	Local Investigator
	FIN06 - Tampere - Department of Pediatrics, Tampere University Hospital	Jason	Eeva	Local Investigator
		Selvenius	Jenni	Local Investigator
		Siljander	Heli	Co-Investigator
	FIN07 - Pori - Satakunta Central Hospital	Ylitalo	Samuli	Local Investigator
	FIN08 - Jyväskylä - Central Finland Central Hospital	Paajanen	Ilkka	Local Investigator

LIST OF TRIGR INVESTIGATORS

Administration/ Country	Study center	Last name	First name	Position
	**FIN09 - Seinäjoki - South Ostrobotnia Central Hospital**	Talvitie	Timo	Local Investigator
	**FIN10 - Hyvinkää - Hyvinkää Hospital**	Lindström	Kaija	Local Investigator
	**FIN11 - Kuopio - Department of Pediatrics, Kuopio University Hospital**	Huopio<br>Pesola	Hanna<br>Jouni	Local investigator<br>Co-Investigator
	**FIN12 - Oulu - Department of Pediatrics, Oulu University Hospital**	Veijola<br>Tapanainen	Riitta<br>Päivi	Local Investigator<br>Co-Investigator
	**FIN13 - Hämeenlinna - Kanta-Hame Central Hospital**	Alar	Abram	Local Investigator
	**FIN14 - Vaasa - Vaasa Central Hospital**	Popov	Erik	Local Investigator
	**FIN15 - Lappeenranta - South Carelian Central Hospital**	Virransalo	Ritva	Local Investigator
	**FIN16 - Mikkeli - Mikkeli Central Hospital**	Nykänen	Päivi	Local Investigator

LIST OF TRIGR INVESTIGATORS

Administration/ Country	Study center	Last name	First name	Position
**Germany**	**GER01 - Hannover - Kinder- und Jugendkrankenhaus – Auf der Bult**	Aschemeier	Bärbel	National Coordinator
		Danne	Thomas	National Investigator
		Kordonouri	Olga	Co-Investigator
**Hungary**	**HUN01 - Budapest - Semmelweis Medical University**	Krikovszky	Dóra	Co-Investigator
		Madácsy	László	National Investigator
**Italy**	**ITA01 - Rome - University Campus Bio-Medico of Rome**	Khazrai	Yeganeh Manon	Local Coordinator
		Maddaloni	Ernesto	Local Coordinator
		Pozzilli	Paolo	National Investigator
	**SAR01 - Cagliari - St. Michele Hospital**	Mannu	Carla	Local Coordinator
		Songini	Marco	National Investigator
**Luxembourg**	**LUX01 - Luxembourg - Centre Hospitalier de Luxembourg**	de Beaufort	Carine	National Investigator
		Schierloh	Ulrike	Co-Investigator
**The Netherlands**	**NET01 - Rotterdam - Sophia Children's Hospital**	Bruining	Jan † *	National Investigator
		Bisschoff	Margriet	National Coordinator
**Poland**	**POL01 - Wroclaw - Medical University of Wroclaw**	Basiak	Aleksander	Co-Investigator
		Wasikowa	Renata	National Investigator
	**POL02 - Krakow - Polish-American Children's Hospital**	Ciechanowska	Marta	Local Investigator
	**POL03 - Katowice - Medical University of**	Deja	Grazyna	Co-Investigator
		Jarosz-Chobot	Przemyslawa	Local Investigator

LIST OF TRIGR INVESTIGATORS

Administration/ Country	Study center	Last name	First name	Position
	**Silesia**			
	**POL04 - Lodz - Medical University of Lodz**	Szadkowska	Agnieszka	Co-Investigator
	**POL05 - Lodz - Polish Mother's Memorial Hospital (I.C.Z.M.P)**	Cypryk<br>Zawodniak-Szalapska	Katarzyna<br>Malgorzata	Local Investigator<br>Co-Investigator
**Spain**	**SPA01 - Cruces University Hospital-UPV/EHU-CIBERDEM/CIBERER, Barakaldo, Spain**	Castano<br>Chueca<br>Gonzalez Frutos	Luis<br>Maria<br>Teba	National Investigator<br>Co-Investigator<br>Local Coordinator
	**SPA02 - Madrid - Hospital Clinico San Carlos**	Serrano-Ríos<br>Martínez-Larrad<br>Hawkins	Manuel † *<br>María Teresa<br>Federico Gustavo	National Investigator<br>Local Coordinator<br>Co-Investigator
	**SPA03 - Madrid - Hospital Gregorio Marañon**	Rodriguez Arnau	Dolores	Co-Investigator
**Sweden**	**SWE01 - Linköping - University of Linköping**	Ludvigsson<br>Smolinska Konefal	Johnny<br>Malgorzata	National Investigator<br>National Coordinator
	**SWE02 - Uddevalla - Uddevalla Hospital**	Hanas	Ragnar	Local Investigator
	**SWE03 - Göteborg - GothenburgThe Queen Silvia Children's Hospital**	Lindblad	Bengt	Local Investigator

LIST OF TRIGR INVESTIGATORS

Administration/ Country	Study center	Last name	First name	Position
	**SWE05 - Halmstad - Halmstad Hospital**	Nilsson	Nils-Östen	Local Investigator
	**SWE06 - Trollhättan - Trollhättan Hospital**	Fors	Hans	Local Investigator
	**SWE07 - Norrköping - Vrinnevi Hospital**	Nordwall	Maria	Local Investigator
	**SWE08 - Borås - Borås Hospital**	Lindh	Agne	Local Investigator
	**SWE09 - Karskrona - Karlskrona Hospital**	Edenwall	Hans	Local Investigator
	**SWE10 - Örebro - University Hospital**	Åman	Jan	Local Investigator
	**SWE11 - Jönköping - Ryhovs Hospital**	Johansson	Calle	Local Investigator
**Switzerland**	**SWT01 - Zürich - University Children's Hospital**	Gadient	Margrit	Local Coordinator
		Konrad	Daniel	National Investigator
		Schoenle	Eugen	National Investigator
**USA**	**USA01 – Pittsburgh, Pennsylvania - Children's Hospital of Pittsburgh**	Becker	Dorothy	USA National Investigator / Pittsburgh Local Investigator
		Daftary	Ashi	Co-Investigator
		Klein	Mary Beth	Pittsburgh Coordinator
		Gilmour	Carol	Co-Investigator

LIST OF TRIGR INVESTIGATORS

Administration/ Country	Study center	Last name	First name	Position
	**USA02 – Seattle, Washington - VA Puget Sound Health Care System and University of Washington**	Palmer	Jerry	Local Investigator
		Palmer	Patty	Local Investigator
		Malone	Patty	Coordinator
	**USA03 - St. Louis, Missouri - Washington University**	Tanner-Blasiar	Marilyn	Coordinator
		White	Neil	Local Investigator
	**USA04 - Los Angeles, California - Mattel Children's Hospital of UCLA**	Devaskar	Uday	Local Investigator
		Horowitz	Heather	Coordinator/dietitian
		Rogers	Lisa	Coordinator/dietitian
	**USA05 – Ponce, Puerto Rico - Ponce School of Medicine**	Colon	Roxana	Coordinator
		Frazer	Teresa	Co-Investigator
		Torres	Jose	Local Investigator
	**USA06 - New York, New York - Naomie Berrie Diabetes Center**	Goland	Robin	Local Investigator
		Greenberg	Ellen	Coordinator
		Schachner	Holly	Co-Investigator
		Softness	Barney	Co-Investigator
**Laboratories**	**HLA-typing Laboratory – University of Turku, Turku – Finland**	Ilonen	Jorma	Head of HLA-typing Laboratory
	**HLA-typing Laboratory – University of Pittsburgh, Pennsylvania - USA**	Trucco	Massimo	Head of HLA-typing Laboratory
		Nichol	Lynn	Chief Technician

LIST OF TRIGR INVESTIGATORS

Administration/ Country	Study center	Last name	First name	Position
	**Cow's Milk Antibody Laboratory – University of Helsinki, Helsinki – Finland**	Savilahti	Erkki	Head of Cow's Milk Antibody Laboratory
	**Autoantibody Laboratory – University of Helsinki, Helsinki – Finland**	Härkönen	Taina	Co-Investigator
		Knip	Mikael	Head of Antibody Laboratory
	**T-Cell Laboratory – Helsinki, Helsinki – Finland**	Vaarala	Outi	Head of T-cell Laboratory
		Luopajärvi	Kristiina	Co-Investigator
	**T-Cell Laboratory – Hospital for Sick Children, Toronto, Ontario - Canada**	Dosch	Hans-Michael	Head of T-Cell Laboratory

† * Deceased
